# ROP GTPase-Dependent Actin Microfilaments Promote PIN1 Polarization by Localized Inhibition of Clathrin-Dependent Endocytosis

**DOI:** 10.1371/journal.pbio.1001299

**Published:** 2012-04-03

**Authors:** Shingo Nagawa, Tongda Xu, Deshu Lin, Pankaj Dhonukshe, Xingxing Zhang, Jiri Friml, Ben Scheres, Ying Fu, Zhenbiao Yang

**Affiliations:** 1 Center for Plant Cell Biology, Department of Botany and Plant Sciences, University of California, Riverside, California, United States of America; 2 Temasek Lifesciences Laboratory Ltd, National University of Singapore, Singapore; 3 State Key Laboratory of Plant Physiology and Biochemistry, Department of Plant Sciences, College of Biological Sciences, China Agricultural University, Beijing, China; 4 Department of Biology, Faculty of Science, Utrecht University, Utrecht, The Netherlands; 5 Department of Plant Systems Biology, VIB and Department of Plant Biotechnology and Genetics, Ghent University, Ghent, Belgium; University of California, San Diego, United States of America

## Abstract

Cell polarization via asymmetrical distribution of structures or molecules is essential for diverse cellular functions and development of organisms, but how polarity is developmentally controlled has been poorly understood. In plants, the asymmetrical distribution of the PIN-FORMED (PIN) proteins involved in the cellular efflux of the quintessential phytohormone auxin plays a central role in developmental patterning, morphogenesis, and differential growth. Recently we showed that auxin promotes cell interdigitation by activating the Rho family ROP GTPases in leaf epidermal pavement cells. Here we found that auxin activation of the ROP2 signaling pathway regulates the asymmetric distribution of PIN1 by inhibiting its endocytosis. ROP2 inhibits PIN1 endocytosis via the accumulation of cortical actin microfilaments induced by the ROP2 effector protein RIC4. Our findings suggest a link between the developmental auxin signal and polar PIN1 distribution via Rho-dependent cytoskeletal reorganization and reveal the conservation of a design principle for cell polarization that is based on Rho GTPase-mediated inhibition of endocytosis.

## Abstract 2

A study in leaf epidermal pavement cells reveals that auxin activation of a Rho-like GTPase from plants induces inhibition of endocytosis through the clathrin-mediated pathway by regulating the accumulation of cortical F-actin.

## Author Summary

Formation of cell polarity is a process of distributing cellular structures or molecules in an asymmetric manner. This process plays an important role in the generation of diverse cell forms and types. In plants, the quintessential hormone auxin is important for diverse physiological functions, including growth and development of cells and organs. To perform these functions, auxin must be transported and localized to specific regions within the plant. This is partially mediated by polar distribution of the PIN-FORMED (PIN) auxin efflux transporters, which transport auxin outside of the cell and allow for the directional short- and long-distance transport of auxin throughout plant tissues and organs. Although auxin itself has been implicated as a signal to regulate PIN polar distribution, how auxin does so remains to be elucidated. We previously showed that auxin promotes the generation of “puzzle-piece” polarity in leaf epidermal pavement cells, which contain interdigitated lobes and indentations, by activating the ROP (Rho-like GTPases from plants) members of the conserved Rho family of small GTPases. Here, we find that auxin-dependent local activation of ROP2 in the lobe region inhibits PIN1 internalization into the endosomal compartments (or endocytosis), leaving higher levels of PIN1 polar distribution in the lobe region. PIN1 internalization is inhibited by altering the actin cytoskeleton through the ROP2 effector protein RIC4, a protein involved in cytoskeletal remodeling. On the basis of our findings, we propose that the Rho GTPase-mediated inhibition of endocytosis of PIN1 provides a self-organizing mechanism for the polar PIN1 distribution. Rho GTPase-based inhibition of endocytosis is also important for the formation of cell polarity in animal cells. Thus, we conclude that Rho GTPase signaling to inhibit endocytosis is a common mechanism for cell polarization in multicellular organisms.

## Introduction

Cell polarity is a conserved cellular property that is necessary for the generation of diverse forms and types of cells in both uni- and multicellular organisms [1],[2]. The general design principles that govern the formation of polarity and how they are used to generate diverse forms of polarity is a fundamental issue of developmental mechanisms. In the unicellular yeast, Rho family GTPase-mediated activation of endocytosis is required for cell polarization [3]–[5]. In contrast, emerging evidence suggests that Rho family GTPase-mediated inhibition of endocytosis is essential for the polarization of cells in some multicellular tissues as shown in cultured epithelial cells from rat [6] and neuroectodermal epithelial cells from *Drosophila* [7]. It is unclear whether Rho-mediated inhibition of endocytosis is a common design principle for polarity establishment in multicellular systems and how the inhibition of endocytosis is regulated.

In multicellular plants, coordinated polarization of the proposed auxin efflux carriers PIN-FORMED (PIN) proteins within a plant tissue is required for polar auxin transport and formation of auxin gradients, which regulate a wide range of morphogenetic and growth patterns in plants [8]–[11]. Asymmetric endocytosis and recycling of plasma membrane (PM)-localized PINs have been shown to contribute to the polar PIN localization [12],[13], and auxin has been implicated as a self-organizing signal to polarize PIN proteins through its inhibition of clathrin-dependent PIN endocytosis in root cells, which is mediated by the auxin-binding protein 1 (ABP1) putative cell surface auxin receptor [14],[15]. We studied auxin regulation of cell polarity formation and PIN1 polarization in *Arabidopsis* leaf epidermal pavement cells (PCs), which display multipolarity by forming the puzzle-piece appearance with interdigitated lobes and indentations [16]–[20]. Recently we showed that ABP1-dependent auxin signaling promotes the formation of multipolarity in PCs by activating Rho-like GTPases from plants (ROPs) that are associated with the plasma membrane [19],[21]. ROPs also regulate other processes mediated by auxin such as root hair development, lateral root formation, and root gravitropic responses [22]–[24]. In addition, auxin activation of ROPs is associated with auxin regulation of gene expression in the nucleus [25],[26].

We found that polar PIN1 localization to the tip of lobes in PCs is dependent upon ROP2, which is activated by auxin in the same PM region where PIN1 is localized [19]. PIN1 is required for ROP2 activation and lobe formation, supporting a role for auxin in self-organizing PIN1 polarization in PCs [19]. How auxin-activated ROP2 regulates PIN1 polarization is unknown. One possible mechanism would be the inhibition of PIN1 endocytosis by activated ROP2, because inactivation of ROP2 leads to PIN1 internalization in PCs [19]. This finding is consistent with the report showing that the expression of constitutively active ROPs inhibited internalization of the endocytosis tracer dye FM-64 in roots and guard cells [27]–[29]. ROP2 regulates the formation of the multipolarity in PCs by activating RIC4 [17], a member of the ROP INTERACTIVE CRIB MOTIF-CONTAINING proteins (RICs) family of ROP effector proteins [30]. RIC4 promotes the local accumulation of fine cortical actin microfilaments in the tip of PCs and pollen tubes [17],[31], and actin dynamics has been implicated in the regulation of auxin transport and PIN endocytosis [32]–[34]. These observations raise an interesting possibility that the ROP2-RIC4 pathway could regulate PIN1 polarization through endocytic trafficking and the actin cytoskeleton.

In this report we show that PIN1 endocytosis is preferentially inhibited in the PM region of lobes and that auxin activation of ROP2 in this region inhibits clathrin-dependent PIN1 endocytosis, allowing PIN1 to be polarized to the ROP2-active region. We further demonstrate that ROP2 promotion of F-actin accumulation via its effector protein RIC4 is responsible for its inhibition of PIN1 endocytosis. Our results reveal the conservation of a new design principle for cell polarization, which is based on localized inhibition of endocytosis by Rho GTPase signaling in multicellular plants and animals, and provide new insights into the mechanisms by which Rho GTPases inhibit clathrin-dependent endocytosis of polarity proteins. Our results establish an auxin signaling pathway leading to the polarization of PIN proteins that is essential for pattern formation and morphogenesis in multicellular plants.

## Results

### ROP2 Inhibits PIN1 Endocytosis in the Lobe Region of PCs

**Figure 1:**
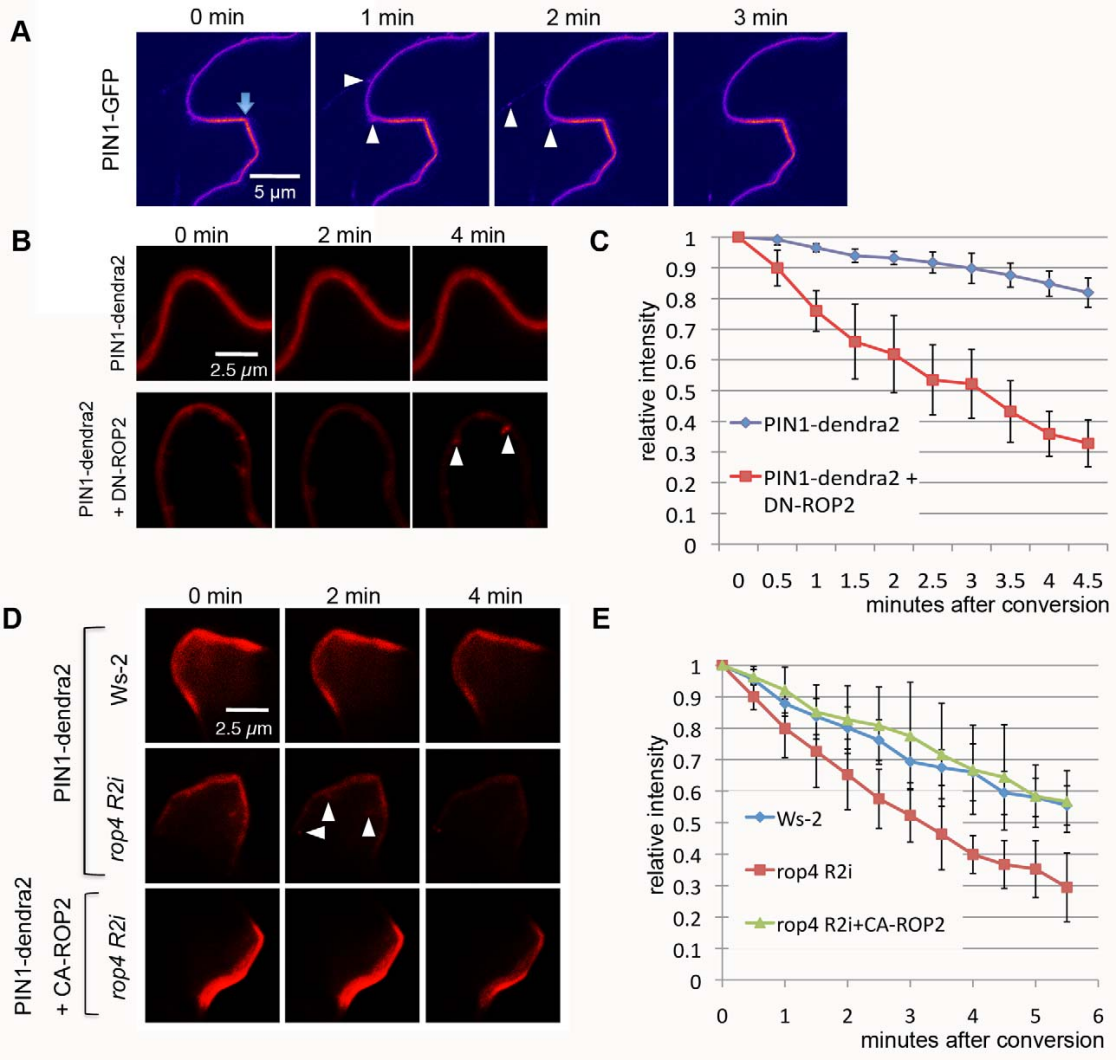
PIN1 endocytosis is inhibited in the lobe region by the ROP2 pathway in PCs. (A) Time-lapse imaging of PIN1-GFP transiently expressed in tobacco PCs. PIN1-GFP signal was imaged using laser scanning confocal microscopy 3 d after the infiltration of tobacco leaves with agrobacterium containing *pPIN1-PIN1-GFP*. Internalization of PIN1-GFP from the PM (arrowheads) occurred preferentially in the indention region but not in the lobe region (arrow) where stronger PM accumulation of PIN1-GFP was observed. Note that only a cell at the left side of the image expressed PIN1-GFP, and internalization events were only visualized in that cell but not in the cell at the right side of the images. (B) A time-course analysis of PIN1-dendra2 internalization after photo-conversion in tobacco PCs. Signal in the red color represents photo-converted PIN1-dendra2. PIN1-dendra2 was transiently expressed in tobacco PCs for 3 d before photo-conversion was achieved. Coexpression of DN-ROP2 promoted vesicle formation (arrowheads) from the PM and accelerated decrease of PM signal. (C) Quantitative analysis of the PM signal representing photo-converted PIN1-dendra2 shown in (B). Relative intensity was measured as absolute value of intensity divided by value of intensity in the first time point. Error bars represent standard deviation (SD) (*n* = 5). (D) A time-course analysis of PIN1-dendra2 internalization after photo-conversion in *Arabidopsis* PCs. PIN1-dendra2 (and CA-ROP2) was transiently expressed in WT or *rop4 R2i Arabidopsis* PCs for 24 h. Formation of vesicles (arrowheads) and decrease of PM signal were accelerated in *rop4 R2i* cells. Coexpressing CA-ROP2 suppressed both vesicle formation and decrease of PM signal in *rop4 R2i* cells (*n* = 5). (E) Quantitative analysis of PM signal shown in (D). Measurements of signal were done as in (C).

To test the auxin-mediated self-organizing PIN1 polarization, we investigated how auxin-activated ROP2 signaling regulates PIN1 localization to the lobe tip. We first utilized PIN1-green fluorescent protein (GFP) transient expression in leaves of *Nicotiana benthamiana* (tobacco) plants by the agrobacterium infiltration method [35]. This system allows determining the effect of mutant ROP2 on PIN1-GFP localization independent of PC shape changes, which occur in *Arabidopsis rop2* mutants [16],[17]. Within 3 d after infiltration, PIN1-GFP was detected in PCs of tobacco leaves and localized to the PM with stronger accumulation at the tips of lobes as in *Arabidopsis* PCs (Figure 1A, arrow). PIN1-GFP signal was also observed in the cytoplasm as endosome-like vesicles (Figure 1A, arrow). Time-lapse imaging showed that PIN1-GFP appeared to be internalized preferentially in the indentation region but not in the lobe region where stronger PM accumulation of PIN1-GFP was observed (Figures 1A, S1A, and S1B). Both PIN1-GFP and FM4-64 were internalized simultaneously and became colocalized in the same vesicles, confirming that GFP-PIN1 was internalized through endocytosis (Figure S1C).

Because PIN1 internalization appears to occur preferentially in the indentation region but not in the lobe region where ROP2 is activated [17],[19], we hypothesized that ROP2 activation may inhibit endocytosis of PIN1, allowing PIN1 to be polarized in that region. To visualize PIN1 internalization, we utilized PIN1 fused with the dendra2 photo-convertible fluorescent protein (Figures S2 and S3) [36]. Photo-conversion of PIN1-dendra2 transiently expressed in tobacco or *Arabidopsis* leaves was conducted using transient high dosage of irradiation with 405-nm laser (Figures S2A and S3A). To confirm whether PIN1-dendra2 expressed in leaves was internalized from the PM, PIN1-dendra2 cells were treated with Brefeldin A (BFA), which inhibits ADP ribosylation factor (ARF) GEF and arrests endosomal recycling, causing internalized PIN1 to accumulate in an aggregate known as BFA bodies in plant cells [14],[32]. PIN1-dendra2 at the PM was photo-converted from green to red emission. 30 min after photo-conversion, converted PIN1-dendra2 was observed in BFA bodies, which demonstrated the occurrence of PIN1-dendra2 endocytosis (Figure S2A). To test the effect of ROP2 on PIN1-dendra2 endocytosis, we coexpressed a dominant-negative mutant of ROP2 (DN-ROP2) with PIN1-dendra2 and observed the internalization of the photo-converted signal at the PM. In the lobe regions of PCs expressing PIN1-dendra2 only, PIN1-dendra2 vesicles were rarely formed from the PM (Figure 1B). In contrast, in cells expressing both PIN1-dendra2 and DN-ROP2, numerous PIN1-dendra2 vesicles were formed and pinched off from the PM (Figure 1B, arrowheads). Furthermore, time-lapse imaging showed that DN-ROP2 expression greatly accelerated the decrease in the photo-converted PM signal, which was quantified by changes in the relative intensity (Figure 1C) or in the absolute intensity (Figure S2B) of the converted signal. In cells coexpressing DN-ROP2 and PIN1-dendra2, the PM PIN1-dendra2 signal was generally weaker compared to cells expressing PIN1-dendra2 alone (Figure 1B). This finding was likely due to the DN-ROP2–mediated induction of endocytosis, but not its general toxic effect, because DN-ROP2 expression did not affect the expression and localization patterns of several endosomal markers (Figure S4). Thus, these results show that DN-ROP2 expression promoted PIN1-dendra2 internalization.

To confirm that the effect of DN-ROP2 on PIN1 endocytosis in tobacco cells reflected the function of ROP2 in *Arabidopsis*, we transiently expressed PIN1-dendra2 in the PCs of wild type (WT) or the *rop4-1 rop2* RNAi line, in which ROP2 is down-regulated by RNAi and the functionally redundant ROP4 is knocked out (*rop4 R2i*) [17]. As expected, photo-converted signal was found in vesicles budding from the PM and decreased rapidly from PM in *rop4 R2i* cells but not in WT control cells (Figures 1D, 1E, and S3B). Moreover, expressing the constitutive active form of ROP2 (CA-ROP2) in *rop4 R2i* cells suppressed PIN1-dendra2 internalization (Figures 1E and S3B). These results indicate that ROP2/ROP4 suppresses PIN1 internalization, which supports our hypothesis that active ROP2 inhibits PIN1 endocytosis in the lobe region.

**Figure 2:**
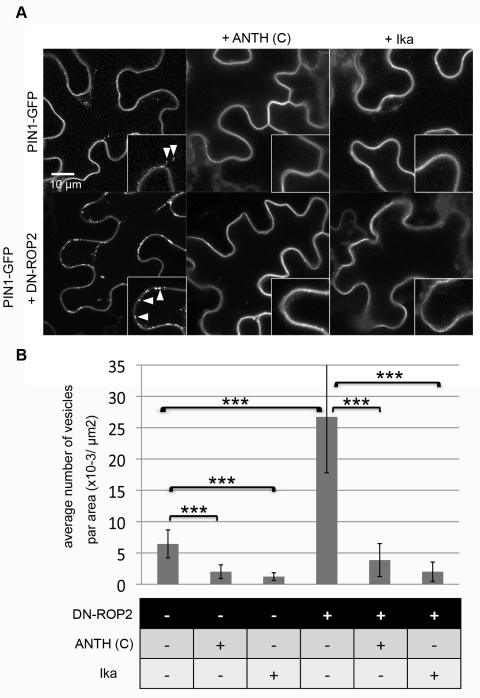
ROP2 regulates clathrin-dependent PIN1 endocytosis in PCs. PIN1-GFP was transiently expressed or coexpressed with DN-ROP2 and/or ANTH(C) constructs in tobacco PCs for 3 d and visualized by confocal microscopy. DN-ROP2 and/or ANTH(C) expression was induced by DEX treatment for 6 h before observation. Ika (5 µM) treatment was carried out by infiltration 2 h before observation. (A) Internalization of PIN1-GFP into vesicles (arrowheads) was observed in control (upper left) and was greatly enhanced by DN-ROP2 expression (lower left). The formation of these PIN1-GFP vesicles was inhibited by the expression of ANTH(C) (middle panels) or treatment with Ika (right panels) in both control (upper panels) and DN-ROP2 cells (lower panels). (B) Quantitative analysis of vesicle numbers shown in (A). Medial sections of confocal scanning images were taken from ten different leaves in at least three independent experiments, and the mean number of vesicles per area of cells was determined. Error bars represent SD (*n* = 10). *p*-Values were determined by two-tailed Student's *t* test assuming equal variances (***, *p*<0.001).

We next tested the identity of the PIN1 vesicles induced by DN-ROP2 expression by examining the colocalization with known endocytic markers in plants. Coexpression of DN-ROP2 with PIN1-GFP in tobacco leaves greatly increased the number of PIN1-GFP vesicles in the cytoplasm (Figure 2A and 2B), similar to the PIN1-dendra2 vesicles. Previous studies showed that endocytic trafficking mediated by the Rab5 family of GTPases plays an essential role in various developmental processes including PIN polarization [13],[37],[38]. Ara7, a Rab5 homolog, resides in an endosomal compartment from which various internalized proteins, such as PIN1, are sorted for targeting to vacuoles or recycling to the PM [39]. In cells coexpressing Venus-Ara7, PIN1-GFP, and DN-ROP2, most PIN1-GFP vesicles overlapped with Venus-Ara7 (Figure S5). Thus, most PIN1 vesicles induced by DN-ROP2 were localized to the endosomal compartment containing Ara7. Taken together our results suggest that activated ROP2 in the lobe region inhibits PIN1 endocytosis in that region.

### ROP2 Inhibits Endocytosis through the Clathrin-Dependent Pathway

Several types of endocytosis have been characterized in yeast or animals [40]. We speculated that the clathrin-dependent endocytic pathway contributed to the PIN1 internalization in PCs because this pathway has been reported to modulate the internalization of PIN proteins in other tissues [15],[41],[42]. To test this notion, we inhibited clathrin-dependent endocytosis by coexpressing the C-terminal region of AtAP180 protein (ANTH[C]) with PIN1-GFP. The conserved AP180 protein contains both the PIP2-binding domain and the clathrin-binding domain and is essential for the early stage of clathrin-dependent endocytosis [43],[44]. ANTH(C), which contains the clathrin-binding domain (ANTH domain), has a dominant-negative effect on the function of AP180 protein and inhibits the clathrin-mediated endocytosis [43]. Overexpression of ANTH(C) greatly reduced the number of PIN1-GFP–associated vesicles and suppressed DN-ROP2 induction of the PIN1-GFP vesicles (Figure 2A and 2B). ANTH(C) did not have a general toxic effect, because its expression did not affect the expression and localization of several other endosomal markers (Figure S4). Treatment with Ikarugamycin (Ika), a specific inhibitor of clathrin-dependent endocytosis [45], produced the same effect as ANTH(C) overexpression (Figure 2A and 2B). These results suggest that ROP2 activation suppressed clathrin-dependent endocytosis of PIN1.

### ROP2 Mediates Auxin-Induced Inhibition of Endocytosis

Because we previously showed that auxin activates ROP2 in the regulation of PC shape formation [19], we next sought to test whether ROP2-mediated inhibition of endocytosis is also regulated by auxin. We first monitored the uptake of FM1-43 in the PCs of WT or *rop4 R2i* plants. BFA treatment for 2 h resulted in the accumulation of the FM dye in aggregated structures (BFA bodies) in WT cells (Figure S6A). Treatments with 5–10 µM auxin inhibits the internalization of FM dyes in root cells [14]. We found that application of naphthalene acetic acid (NAA) as low as 100 nM prevented the accumulation of FM1-43 in BFA bodies (Figure S6A). In *rop4R2i* PCs, FM1-43 accumulated in BFA compartments as in WT cells (Figure S6A). However, NAA did not prevent the accumulation of FM1-43 in these structures in *rop4 R2i* cells (Figure S6A). Furthermore, expression of CA-ROP2 suppressed FM1-43 accumulation in BFA bodies in PCs treated with BFA (Figure S6B). Thus, these results suggest that ROP2 is required for the auxin-induced inhibition of endocytosis.

**Figure 3:**
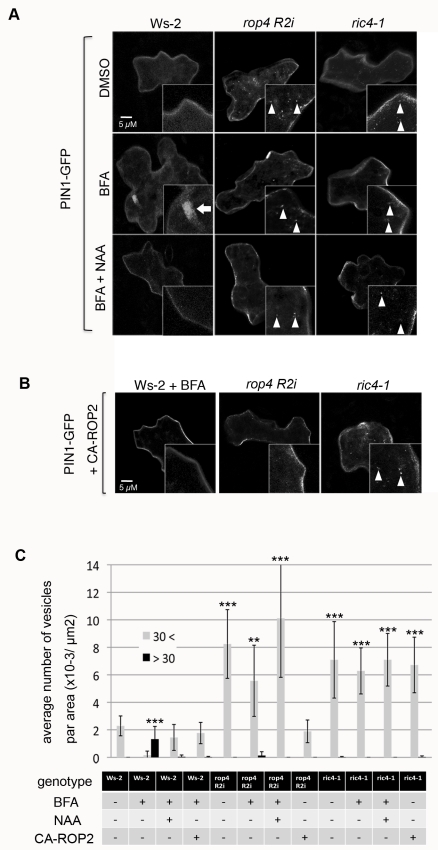
The ROP2-RIC4 pathway mediates auxin-induced inhibition of PIN1 endocytosis. PIN1-GFP was transiently expressed for 24 h alone (A) or coexpressed with CA-ROP2 construct (B) in *Arabidopsis* PCs of WT control (Ws-2), *rop4 R2i*, or *ric4-1* leaves treated with DMSO (mock control), BFA, or BFA plus NAA. Medial sections of confocal images were taken, and internalized PIN1-GFP structures (BFA body-like, larger than 30 pixels; Ara7 vesicle-like, smaller than 30 pixels) were analyzed. (A) PIN1-GFP vesicles (Ara7 vesicle-like, see Figure S4) were rarely found in untreated Ws-2 cells and were abundant in *rop4 R2i* or *ric4-1* cells. BFA (50 µM) treatment induced large aggregation of PIN1-GFP structures into BFA bodies in Ws-2 cells, but did not affect PIN1-GFP vesicles in *rop4 R2i* or *ric4-1* cells. Application of 100 nM NAA (BFA+NAA) inhibited the accumulation of PIN1-GFP in BFA bodies in Ws-2, but did not alter the formation of PIN1-GFP vesicles in *rop4 R2i* or *ric4-1* cells. (B) CA-ROP2 expression prevented PIN1-GFP accumulation in BFA bodies in Ws-2. CA-ROP2 also suppressed the formation of PIN1-GFP vesicles in *rop4 R2i* but not in *ric4-1*. (C) Quantitative analysis of PIN1-GFP containing BFA bodies and vesicles shown in (A) and (B). Vesicles were categorized by pixel area in the images into two classes (see Figure S5): <30 = smaller than 30 pixels, similar to the size of Ara7 vesicles; >30 = larger than 30 pixel, BFA bodies. Error bars represent SD (*n* = 10). *p*-Values (against Ws-2–untreated cells) were determined by two-tailed Student's *t* test assuming equal variances (**, *p*<0.01; ***, *p*<0.001).

Auxin-induced inhibition of PIN1 internalization has been well documented in roots [14],[15],[46]. We next asked whether PIN1 internalization in *Arabidopsis* PCs is also inhibited by auxin in a ROP2-dependent manner by transiently expressing PIN1-GFP in *rop4 R2i* cells (Figure 3) [19]. The BFA-induced PIN1-GFP structures were similar to the BFA compartments containing FM1-43 (Figures 3A and S6A). Treatments with NAA (100 nM) inhibited PIN1-GFP accumulation in the BFA compartments (Figure 3A and 3C). Thus, auxin suppresses PIN1 internalization in PCs as in other tissues. However, NAA treatments did not reverse PIN1-GFP accumulation to endosomal vesicles in *rop4 R2i* cells (Figure 3A and 3C). When CA-ROP2 was coexpressed with PIN1-GFP in *rop4 R2i* cells, the accumulation of PIN1-GFP vesicles was suppressed (Figure 3B and 3C). Therefore, ROP2/4 is required for the inhibitory effect of auxin on PIN1 endocytosis. In contrast to WT cells treated with BFA, PIN1-GFP remained in the endosomal vesicles in *rop4 R2i* cells upon BFA treatment (Figure 3A and 3C), implying that ROP2 may also regulate the PIN1 trafficking from or the transition of these endosomosal vesicles into recycling PIN1 vesicles, which BFA acts on.

### RIC4-Mediated Accumulation of Cortical Actin Microfilaments Is Downstream of ROP2 in the Inhibition of Endocytosis

Actin microfilaments have been implicated in the regulation of polar trafficking of PIN proteins [32],[34],[47],[48], but the exact nature of F-actin and the mechanism by which this F-actin modulates PIN polarization remains elusive. Because ROP2/ROP4 promotes the accumulation of fine cortical actin microfilaments through its downstream target protein RIC4 [17], we assessed whether RIC4-dependent F-actin mediates ROP-dependent PIN1 localization in PCs. We first analyzed the localization of PIN1 in *ric4-1* mutants. Reduction of RIC4 level in *ric4-1* mutants results in abnormalities in the PC shape that is less profound than but similar to those in the loss-of-function ROP2/ROP4 mutants [17]. In PCs of *ric4-1*, PIN1-GFP was internalized into endosomal vesicles (Figure 3A and 3C) as in *rop4 R2i* cells [19], and NAA treatment did not reverse PIN1-GFP accumulation in the endosomal vesicles in *ric4-1* PCs (Figure 3A and 3C). Unlike *rop4 R2i* cells, however, coexpression of CA-ROP2 did not suppress the internalization of PIN1-GFP in *ric4-1* cells (Figure 3A–3C). As shown for *rop4 R2i* cells, NAA treatments did not suppress FM dye accumulation in BFA compartments in *ric4-1* PCs (Figure S6A). Taken together, these results suggest RIC4 acts downstream of ROP2/ROP4 in the suppression of PIN1 endocytosis in PCs.

**Figure 4:**
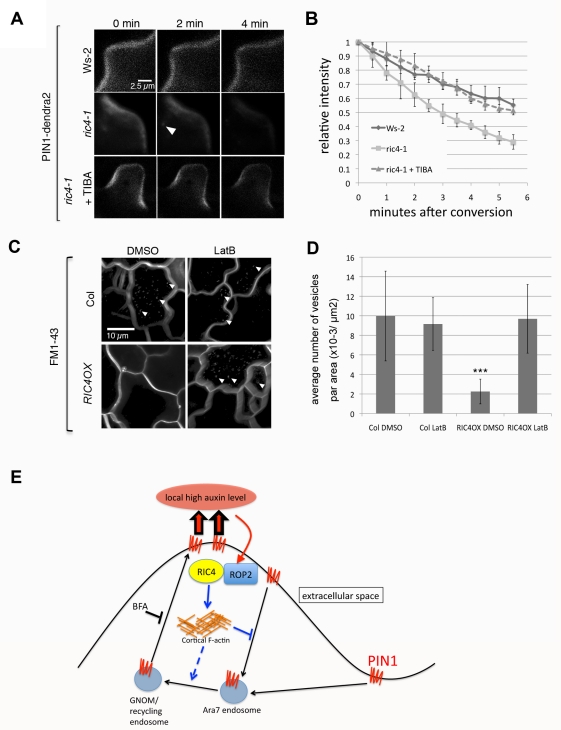
The ROP2-RIC4 pathway inhibits PIN1 endocytosis through the promotion of the accumulation of cortical actin filaments. (A) A time-course analysis of PIN1-dendra2 internalization after photo-conversion in *Arabidopsis* PCs. PIN1-dendra2 was transiently expressed in WT (Ws-2) or *ric4-1* PCs for 24 h. Compared to WT cells, formation of vesicles (arrowheads) and decrease of PM signal were accelerated in *ric4-1* cells as in *rop4 R2i* cells (Figure 1D). Stabilizing F-actin by TIBA treatment for 2 h suppressed both vesicle formation and decrease of PM signal in *ric4-1* cells. (B) Quantitative analysis of PM signal shown in (A). Relative intensity was measured by absolute value of intensity divided by value of intensity in the first image. Error bars represent SD (*n* = 5). (C) An analysis of FM1-43 uptake in Col-0 WT and *RIC4* overexpressing lines. Images were taken 1 h after leaves were incubated in liquid MS medium containing FM1-43. Each image is a stack image of 40 images taken for around 4 min to visualize internalized signal. Vesicles containing FM1-43 (arrowheads) were accumulated in Col-0 but not in *RIC4* overexpressing cells (RIC4 OX). Application of Latrunculin B (100 nM, LatB) induced uptake of FM1-43 into small vesicles in *RIC4 OX* cells. (D) Quantitative analysis of vesicle numbers shown in (C). Images were taken from ten different leaves in at least three independent experiments, and the mean number of vesicles per area of cells was determined. Error bars represent SD (*n* = 30). *p*-Values were determined by two-tailed Student's *t* test assuming equal variances (***, *p*<0.001). (E) A model for a ROP-signaling-based feed-forward mechanism for PIN1 polarization to the lobe region of PCs. Extracellular auxin activates ROP2 in the lobing region, and the activated ROP2-RIC4 pathway inhibits PIN1 internalization through RIC4-dependent cortical F-actin, leading to PIN1 polarization to the lobing region. PIN1-based export of auxin induces further ROP2 activation to complete the feed-forward cycle. In addition to its inhibition of endocytosis, the ROP2-RIC4 pathway is also required for the endosomal trafficking of PIN1 from Ara7-marked endosomes to recycling endosomes.

Given a role for RIC4 in promoting the accumulation of cortical F-actin in the lobe region, we hypothesize that RIC4 inhibits PIN1 endocytosis through the RIC4-dependent F-actin. We tested this hypothesis by using a combination of F-actin–modifying chemicals and genetically modified *Arabidopsis* plants with both loss of and gain of RIC4 function. Stabilization of F-actin by treatments with chemicals such as TIBA or Jasplakinolide is reported to inhibit PIN endocytosis in roots of *Arabidopsis* [33]. Similarly, these chemicals stabilized cortical fine F-actin and inhibited endocytosis in WT *Arabidopsis* PCs, because treatment with TIBA or Jasplakinolide induced accumulation of cortical fine F-actin (Figure S7) and inhibited uptake of FM1-43 (Figure S8). Time-lapse imaging of photo-converted PIN1-dendra2 showed that loss of RIC4 function (*ric4-1*) greatly accelerated the internalization of photo-converted PIN1-dendra2 as expected (Figure 4A and 4B), whereas TIBA treatments completely reversed the acceleration of PIN1-dendra2 induced by the *ric4-1* mutation or DN-ROP2 expression (Figures 4A, 4B, S9A, and S9B). In contrast, *RIC4* overexpression suppressed FM1-43 internalization, as did *CA-ROP2* expression (Figures 4C and S6B). In *RIC4*-overexpressing plants treated with the actin-depolymerizing drug Latrunculin B (100 nM), the accumulation of internalized vesicles was restored (Figure 4C and 4D; arrows). The same concentration of Latrunculin B greatly reduced the amount of the cortical fine F-actin, but not that of cytoplasmic actin cables (Figure S7). These results suggest that the accumulation of the cortical fine F-actin, which is activated by the ROP2-RIC4 pathway in the lobing region, inhibits the endocytosis of PIN1, and consequently promoting PIN1 polarization in the lobing region of the PM in PCs (Figure 4E).

## Discussion

Our findings here have established an auxin-activated ROP2-signaling pathway that regulates PIN1 protein polarization to the PC lobe through the localized inhibition of PIN protein endocytosis. Given the requirement of PIN1 for the ROP2 activation at the lobe region of the PM [19], this signaling pathway underscores a positive feedback loop leading to PIN1 polarization, which provides strong support for the hypothesis that auxin acts as a self-organizing signal in the control of PIN-dependent auxin efflux [14],[15]. Furthermore, we have demonstrated that auxin signaling links the Rho GTPase-dependent accumulation of the cortical fine F-actin to PIN polarization. This finding provides an important insight into the mechanism for the modulation of F-actin reorganization in its regulation of PIN endocytosis and polarization [32],[47],[49]. Several recent studies implicate actin dynamics in the regulation of PIN endocytic recycling. By using transgenic rice plants that express different levels of mouse talin protein, Nick et al. recently showed that dynamics of actin organization and auxin transport efficiency are coupled [34]. Auxin transport inhibitors such as TIBA were shown to induce bundling of actin filaments and inhibit endocytosis, and thus were suggested to affect auxin transport through actin-mediated vesicle trafficking of auxin transport-related proteins [13]. Our data show that the ROP2/RIC4-dependent auxin signaling pathway induces the accumulation of the cortical fine F-actin, which inhibits clathrin-dependent PIN1 endocytosis that leads to PIN1 polarization. The mechanism by which the ROP2-dependent F-actin accumulation inhibits endocytosis needs to be investigated in the future. In yeast, clathrin-dependent endocytosis requires not only Cdc42 GTPase-dependent polymerization of cortical actin patches but also their dynamics. Similarly in pollen tubes both ROP1 GTPase-dependent polymerization and dynamics of tip F-actin are critical for polarized pollen tube growth [50],[51]. Thus it will be interesting to know whether the polymerization of ROP2-mediated F-actin is also important for clathrin-dependent PIN1 endocytosis.

Importantly our findings show that Rho GTPase inhibition of endocytosis is a conserved design principle for the establishment of cell polarity in plants and animal cells. Rac and Cdc42 inhibition of endocytosis has also been shown to be required for cell polarization in cultured epithelial cells from rat [6]. Rho-GTPase mediates the developmental process of neuroectodermal epithelial cells in *Drosophila*, in which endocytosis of apical proteins are inhibited and their trafficking from early endosome to late endosome is promoted by CDC42 [7]. In plants, auxin inhibition of PIN endocytosis has been implicated in the regulation of PIN polarization that is required for auxin gradient formation and auxin flow and the formation of various developmental patterns [13]–[15],[52]. ROPs have been implicated in the regulation of similar developmental processes [22],[53]. ROP2 appears to regulate PIN2 polarization required for gravitropic responses [24]. ABP1 regulates auxin-induced inhibition of PIN1 endocytosis in roots [15], and acts upstream of ROP2 in the activation of the formation of the multipolarity of PCs in leaves [19]. It is reasonable to speculate that the ABP1/ROP2-based auxin signaling modulates PIN endocytosis in various developmental processes in plants. Thus, Rho GTPase regulation of PIN endocytic trafficking may provide a common mechanism for the regulation of PIN protein polarization.

Apart from the localized inhibition of PIN endocytosis, PIN polarization requires polar recycling of internalized PIN proteins [12],[54]–[56]. In addition to its activation of the RIC4-actin pathway that inhibits PIN1 endocytosis, ROP2 signaling may also promote polar recycling of PIN1. In support of this notion, we previously found that *rop4 R2i* PCs show stronger defects in cell shape formation and PIN1 distribution compared to *ric4-1* PCs [17]. Interestingly, mutations in the ICR1 ROP effector protein induce strong defect in PIN polarization in *Arabidopsis* roots and embryos by affecting PIN recycling [55]. ICR1 is structurally unrelated to RICs and was shown not to affect PIN endocytosis [55],[57]. ICR1 interacts with the *Arabidopsis* homolog of SEC3, a component of the conserved exocyst complex that regulates the docking of exocytic vesicles to the PM site of exocytosis [28],[57]. Loss of ICR1 function also induces a strong defect in PC shape formation. Future work should determine whether ICR1 acts as a ROP2 effector to promote PIN1 recycling into the lobe region of the PM in PCs.

Our data suggest that the ROP2-RIC4-actin pathway participates in other aspects of endosomal trafficking in addition to its inhibition of PIN1 endocytosis. In this work, we found that defects in this pathway cause PIN1 to accumulate in an endosomal compartment containing Ara7 but not in BFA bodies. This finding implies that the ROP2-RIC4-actin pathway either is required for PIN1 trafficking to recycling vesicles or inhibits PIN1 trafficking to vacuolar compartments for degradation [54],[58]. In pollen tubes, the ROP1-RIC4-actin pathway regulates exocytosis required for tip growth [51]. It is possible that RIC4 also contributes to exocytic trafficking of PINs through actin-based targeting of recycling vesicles. Further studies will be needed to determine whether auxin activation of ROP signaling coordinates various downstream pathways leading to PIN polarization, such as the RIC4- and ICR1-dependent pathways.

## Materials and Methods

### Plant Materials and Growth Conditions

Seeds of *Arabidopsis* or *N. benthamiana* (tobacco) were surface sterilized by 50% bleach with 0.1% triton X-100 and washed three times with distilled water. *Arabidopsis* plants were grown at 22°C on MS agar plates or in soil with 16-h light/8-h dark cycles. Tobacco plants were grown in soil with the same light cycles. The double-mutant *ROP2RNAi rop4-1*, *ric4-1*, and *CA-ROP2* lines were described previously [16],[17],[19]. For chemical treatment, BFA, NAA, Ika, TIBA, JASP, and LatB were used from 50 mM, 100 µM, 5 mM, 50 mM, 2 mM, and 500 µM stock solutions dissolved in DMSO. DEX applications for induction of gene expression were done by spraying leaves with 3 µM DEX solution.

### Plasmid Construction

Plasmids used for balistics-mediated transient expression were constructed in *pBI221*. *pBI221-CA-ROP2*, *pBI221-DN-ROP2*, and *pBI221-PIN1-GFP* were described previously [16],[19]. Plasmids for DEX-inducible expression were constructed in derivative of *pTA7002* [59], containing gateway cassettes kindly provided by Yuichiro Watanabe in the university of Tokyo. CDS of *DN-ROP2*, *ARA7*, or partial CDS of an AP180 protein (residue 991–1,959 of *At1g05020*) were introduced to *pENTR* or *pDONR*. LR reactions with obtained entry vectors and *pTA7002-Venus-GW* (*ARA7*), *pTA7002-mCherry-GW* (*C-ANTH*), or *pTA7002-SECFP-GW* (*DN-ROP2*) were performed to obtain *pTA7002-Venus-ARA7*, *pTA7002-mCherry-C-ANTH*, or *pTA7002-SECFP-DN-ROP2*. Entry vector for photo-convertible PIN1 (*pDONR-PIN1-dendra2*) was constructed by replacing *GFP* in *PIN1-GFP* amplified from *pPIN1-PIN1-GFP* [60] to dendra2 [36]. LR reactions with obtained entry vector and *pGWB2* [61] or *pBI221-sGFP-gateway* were performed to obtain *pGWB2-PIN1-dendra2* or *pBI221-PIN1-dendra2*.

### Ballistics-Mediated Transient Expression in Leaf Epidermal Cells

Subcellular localization analysis in *Arabidopsis* PCs was done by ballistics-mediated transient expression as described previously [17]. We used 1 µg *pBI221-PIN1-GFP* or *pBI221-PIN1-dendra2* and 0.5 µg *pBI221-CA-ROP2* for particle bombardment. GFP signal was observed 24 h after bombardment by confocal microscopy (Leica SP2 confocal microscope or Zeiss 710 confocal microscopy). Conditions for imaging were set as 488-nm excitation, collecting bandwidth at 500–570 nm for GFP. For quantification of the number of PIN1-GFP vesicles per area, each cell area or vesicle size was measured using ImageJ.

### Agrobacterium-Mediated Transient Expression in Leaf Epidermal Cells

Subcellular localization analysis in tobacco PCs was done by agrobacterium-mediated transient expression in leaf epidermal cells. Infiltration of agrobacterium for transient expression was performed as a standard protocol [62]. Leica SP2 or Zeiss 710 confocal microscopy was used for observation. Conditions for imaging were set as 488-nm excitation, collecting bandwidth at 495–515 nm for GFP, 514-nm excitation, collecting bandwidth at 560–640 nm for YFP, 442-nm excitation, collecting bandwidth at 450–490 nm for CFP, and 560-nm excitation, collecting bandwidth at 600–720 nm for mCherry. Any bleach-through signal among each channel was removed by adjusting the gain in the each channel using the signal in cells expressing single construct infiltrated at the same experiment.

### Dendra2 Photo-Conversion Experiments

For photo-converting PIN1-dendra2 expressed in tobacco PCs, regions of interest were illuminated by 405-nm laser at 5% power and speed set at 5 using Zeiss 710. For photo-converting PIN1-dendra2 expressed in *Arabidopsis* PCs, regions of interest were illuminated by 405-nm laser at 4% power and speed set at 7 using Zeiss 710. Conditions for imaging photo-converted signal were set as 560-nm excitation, collecting bandwidth at 600–720 nm. Quantification of PM signal at lobe region was performed by measuring intensity of PM along outermost cell outline in lobe sites using ImageJ.

### FM Dye Uptake Experiments

FM1-43 dye uptake experiment was performed in liquid MS medium containing 10 µM FM1-43 using 2-d-old seedlings. Conditions for imaging were set as 488-nm excitation, collecting bandwidth at 500–570 nm.

## Supporting Information

Figure S1 **PIN1-GFP internalization is inhibited at the tips of lobe regions and occurs simultaneously with FM4-64 uptake.** (A) A projection image of two adjacent tobacco PCs transiently expressing PIN1-GFP. Internalization of PIN1-GFP from the PM (arrowheads) occurred preferentially in the indention region but not in the lobe region (arrow). (B) Quantification of position for PIN1-GFP internalization events. PIN1-GFP was transiently expressed in tobacco PCs. Each lobe region was divided equally into top, middle, or bottom part as shown in left panel, and internalization events observed in each part in image sets that were taken for 4 min were counted. Right panel shows average number of internalization events quantified from ten image sets. *p*-Values were determined by two-tailed Student's *t* test assuming equal variances (**, *p*<0.01; ***, *p*<0.001). (C) PIN1-GFP was transiently expressed in tobacco PCs. FM4-64 was infiltrated 10 min before observation. PIN1-GFP localized to the PM and cytoplasmic vesicles (arrows), which overlap with FM4-64.
(TIF)

Figure S2 **Photo-conversion of PIN1-dendra2 expressed in a tobacco leaf PCs and subsequent internalization of the converted signal.** PIN1-dendra2 was transiently expressed in tobacco PCs for 3 d and visualized using confocal microscopy. (A) Left panel: merged images of the green channel (495–515 nm) and the red channel (600–720 nm) before photo-conversion. Tobacco cells expressing PIN1-dendra2 was visualized using 488-nm laser and 560-nm laser for excitation prior to photo-conversion. Middle panel: Merged images of the green channel and the red channel after photo-conversion. Photo-conversion of PIN1-dendra2 expressed in tobacco PCs was achieved by irradiation of 405-nm laser, which produced emission detected by red channel. Right panel: A red channel image of photo-converted PIN1-dendra2 expressed in tobacco PCs treated with BFA for 30 min. BFA (50 µM) treatment induced aggregation of photo-converted signal. (B) Quantitative analysis of reduction of photo-converted signal in absolute value. Datasets used for Figure 1C were used for quantification.
(TIF)

Figure S3 **Photo-conversion of PIN1-dendra2 expressed in** ***Arabidopsis*** **PCs.** (A) PIN1-dendra2 construct was introduced into *Arabidopsis* leaf PCs by ballistic-mediated transformation (see main text) and observed 24 h after bombardment. The green emission of PIN1-dendra2 was obtained by irradiating with 488-nm laser prior to photo-conversion. Setting for the green channel and the red channel was set as in Figure S2A. Photo-conversion of PIN1-dendra2 expressed in an *Arabidopsis* leaf PC was achieved by irradiation with 405-nm laser. (B, C) Quantitative analysis of reduction of photo-converted signal in absolute value. Datasets used for Figure 1E (B) or Figure 4B (C) were used for quantification.
(TIF)

Figure S4 **Coexpressing DN-ROP2 or ANTH(C) did not affect localization of endosome markers.** Endosome markers WAVE7 (RHA1; PVC/late endosome) and WAVE13 (VTI12; TGN/early endosome) were transiently expressed in tobacco PCs for 2 d with or without DN-ROP2 or ANTH(C). DN-ROP2 or ANTH(C) expression was induced by DEX treatment 6 h before observation. Neither markers show any noticeable changes upon coexpressing DN-ROP2 or ANTH(C), suggesting that there are no toxic side effect on expressing DN-ROP2 or ANTH(C).
(TIF)

Figure S5 **Colocalization of internalized PIN1-GFP induced by coexpressing DN-ROP2 with Venus-Ara7.** A colocalization analysis of PIN1 and the Ara7 Rab5 GTPase. PIN1-GFP, DN-ROP2 and Venus-Ara7 were transiently expressed in tobacco PCs for 3 d. DN-ROP2 and Venus-Ara7 expression was induced by DEX treatment 6 h before observation. The majority of vesicles containing PIN1-GFP overlapped with Venus-Ara7 containing vesicles.
(TIF)

Figure S6 **The ROP2-RIC4 pathway mediates inhibition of FM dye uptake by NAA.** Leaves of various *Arabidopsis* lines were incubated with FM1-43 for 2 h and treated with DMSO (control), BFA (100 µM), or BFA (100 µM) plus NAA (100 nM) for 90 min. FM1-43 in PCs was imaged using confocal microscopy. (A) BFA induced FM1-43 aggregation into BFA bodies. NAA treatment suppressed FM1-43 accumulation in BFA bodies in Ws-2, but not in *rop4R2i* or *ric4-1*. (B) CA-ROP2 and RIC4OX did not develop BFA body.
(TIF)

Figure S7 **Effect of actin-related drugs on F-actin in PCs monitored by GFP-mTalin.** GFP-mTalin construct was introduced into WT (Col) *Arabidopsis* leaf PCs by ballistic-mediated transformation and observed 4 h after bombardment. Leaves were treated with DMSO, TIBA (50 µM), JASP (2 µM), or LatB (100 nM) for 1 h. Control cells showed the accumulation of cortical fine F-actin (arrows) preferentially at apparently growing lobes in 84% of cells observed (*n* = 31). Treatments with TIBA or JASP induced broader distribution of cortical fine F-actin in the cortical region in 83% of cells observed (*n* = 30) for TIBA or in 80% of cells observed (*n* = 30) for JASP, suggesting that these treatments induced the stabilization of cortical fine F-actin. Conversely, cells treated with LatB were devoid of cortical fine F-actin in 94% of cells observed (*n* = 31), although thick bundled actin cables were evident in these cells, suggesting the treatment specifically abolished cortical fine F-actin.
(TIF)

Figure S8 **Auxin and actin-stabilizing drugs inhibit FM dye uptake in** ***Arabidopsis*** **PCs.** WT (Ws-2) *Arabidopsis* leaves were treated either DMSO, NAA, TIBA, or JASP simultaneously with FM1-43 for 1 h. Each image is a stack image of 40 images taken for around 4 min to visualize internalized signal. FM1-43 accumulated in vesicles (arrowheads) in DMSO control cells, whereas NAA (100 nM) and antistabilizing drugs (TIBA, 50 µM) and (JASP, 2 µM) suppressed the accumulation of FM1-43 in these vesicles.
(TIF)

Figure S9 **DN-ROP2–induced internalization of PIN1-dendra2 is suppressed by actin stabilization.** PIN1-dendra2 was transiently expressed alone or with DN-ROP2 construct in tobacco leaf PCs for 3 d. Treatments with the TIBA actin-stabilizing drug were as described in Figure S7, and photo-conversion was conducted as described in Figure 1B. This experiment was conducted in conjunction with the one described in Figure 1B. (A) A time-course analysis of PIN1-dendra2 internalization after photo-conversion in tobacco PCs (images of PIN1-dendra2 or PIN1-dendra2+DN-ROP2 are the same images as those used in Figure 1B). TIBA (50 µM) treatment suppressed both vesicle formation and decrease of PM signal induced by DN-ROP2 expression. (B) Quantitative analysis of PM signal shown in (A). Measurements of signal were done as in Figure 1C.
(TIF)

## Abbreviations

ABP1: auxin-binding protein 1
ANTH(C): C-terminal region of AtAP180 protein
BFA: Brefeldin A
CA-ROP2: constitutive active form of ROP2
DN-ROP2: dominant-negative mutant of ROP2
GFP: green fluorescent protein
Ika: Ikarugamycin
NAA: naphthalene acetic acid
PC: pavement cell
PIN: PIN-FORMED
RIC: ROP INTERACTIVE CRIB MOTIF-CONTAINING PROTEIN
ROP: Rho-like GTPases from plants
SD: standard deviation
WT: wild type
