# Supplementary figures and images for: ROP GTPase-Dependent Actin Microfilaments Promote PIN1 Polarization by Localized Inhibition of Clathrin-Dependent Endocytosis
Source: PLoS Biol. 2012 Apr 3;10(4):e1001299. doi: 10.1371/journal.pbio.1001299 (PMC3317906; doi:10.1371/journal.pbio.1001299)

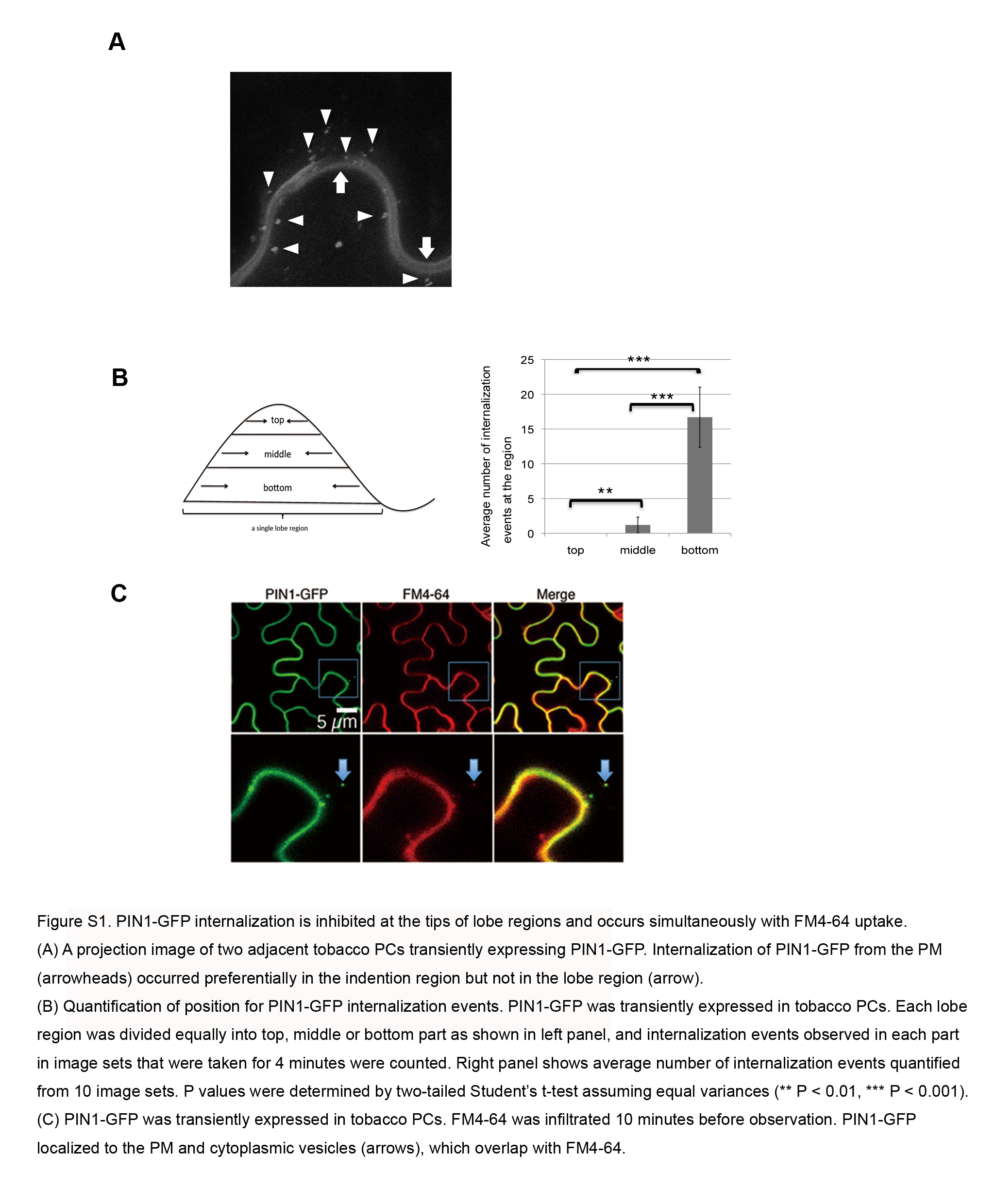

Supplement: Figure S1 — PIN1-GFP internalization is inhibited at the tips of lobe regions and occurs simultaneously with FM4-64 uptake. (A) A projection image of two adjacent tobacco PCs transiently expressing PIN1-GFP. Internalization of PIN1-GFP from the PM (arrowheads) occurred preferentially in the indention region but not in the lobe region (arrow). (B) Quantification of position for PIN1-GFP internalization events. PIN1-GFP was transiently expressed in tobacco PCs. Each lobe region was divided equally into top, middle, or bottom part as shown in left panel, and internalization events observed in each part in image sets that were taken for 4 min were counted. Right panel shows average number of internalization events quantified from ten image sets. p-Values were determined by two-tailed Student's t test assuming equal variances (**, p<0.01; ***, p<0.001). (C) PIN1-GFP was transiently expressed in tobacco PCs. FM4-64 was infiltrated 10 min before observation. PIN1-GFP localized to the PM and cytoplasmic vesicles (arrows), which overlap with FM4-64. (TIF) [file pbio.1001299.s001.tif]

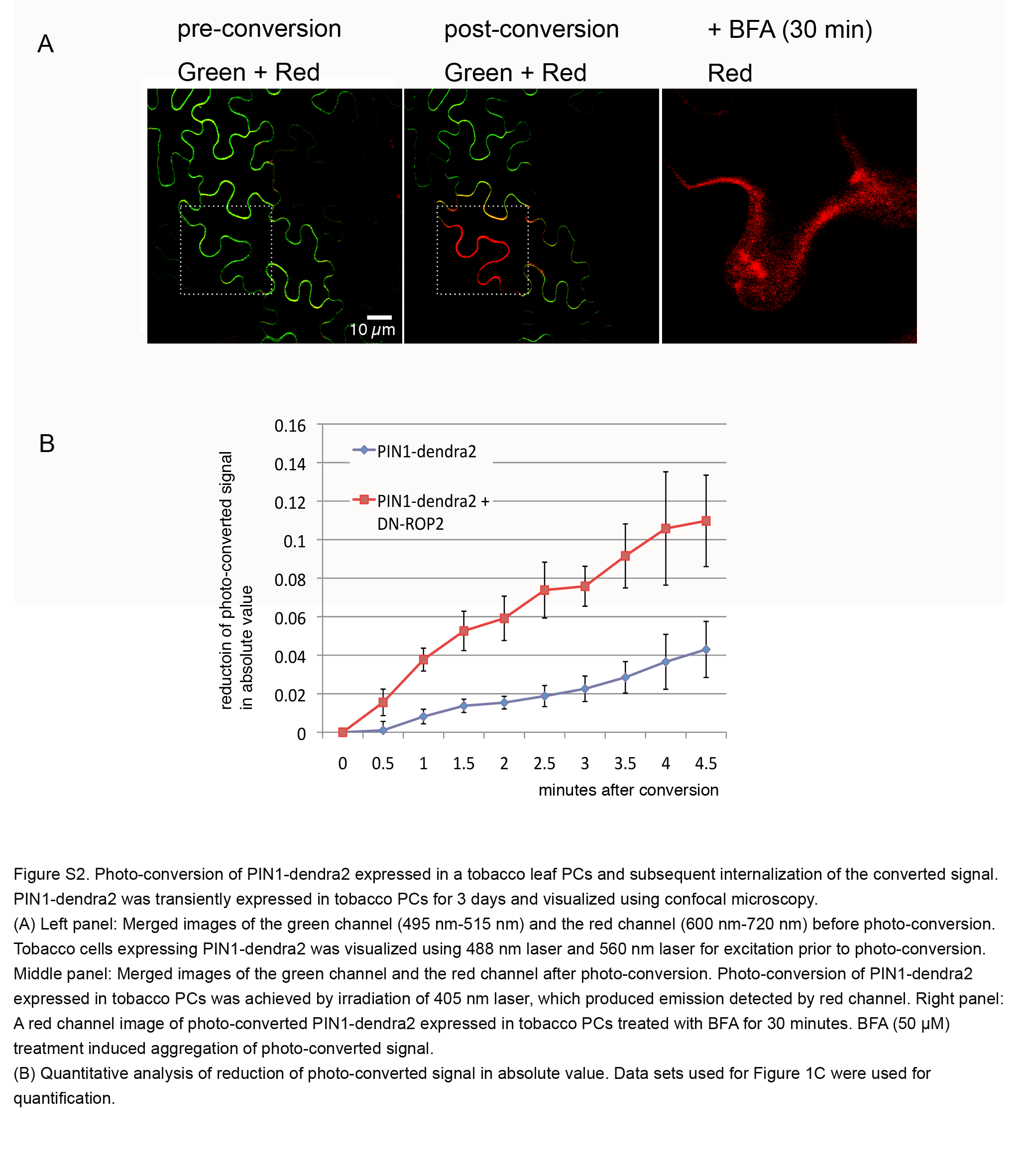

Supplement: Figure S2 — Photo-conversion of PIN1-dendra2 expressed in a tobacco leaf PCs and subsequent internalization of the converted signal. PIN1-dendra2 was transiently expressed in tobacco PCs for 3 d and visualized using confocal microscopy. (A) Left panel: merged images of the green channel (495–515 nm) and the red channel (600–720 nm) before photo-conversion. Tobacco cells expressing PIN1-dendra2 was visualized using 488-nm laser and 560-nm laser for excitation prior to photo-conversion. Middle panel: Merged images of the green channel and the red channel after photo-conversion. Photo-conversion of PIN1-dendra2 expressed in tobacco PCs was achieved by irradiation of 405-nm laser, which produced emission detected by red channel. Right panel: A red channel image of photo-converted PIN1-dendra2 expressed in tobacco PCs treated with BFA for 30 min. BFA (50 µM) treatment induced aggregation of photo-converted signal. (B) Quantitative analysis of reduction of photo-converted signal in absolute value. Datasets used for Figure 1C were used for quantification. (TIF) [file pbio.1001299.s002.tif]

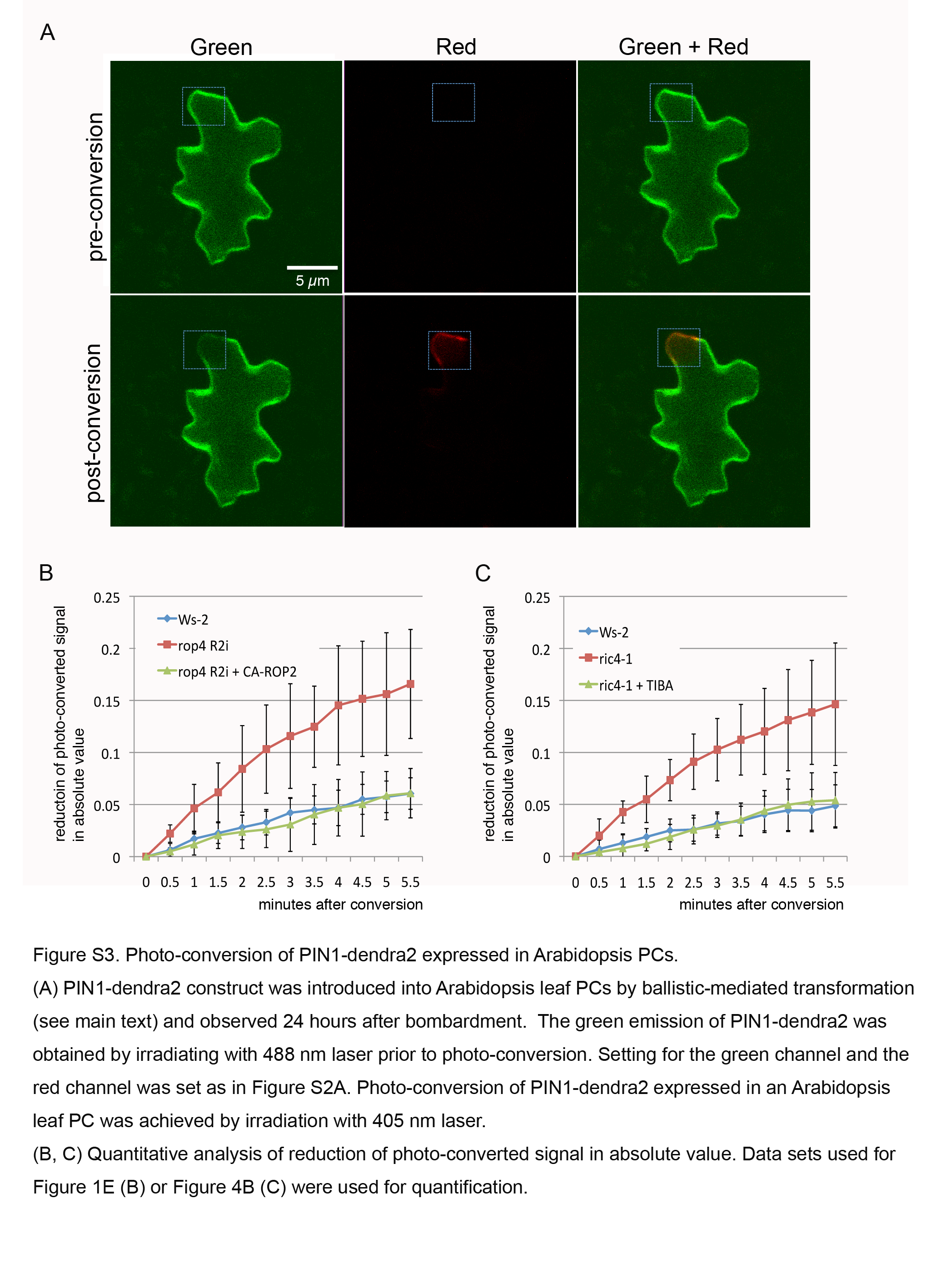

Supplement: Figure S3 — Photo-conversion of PIN1-dendra2 expressed in Arabidopsis PCs. (A) PIN1-dendra2 construct was introduced into Arabidopsis leaf PCs by ballistic-mediated transformation (see main text) and observed 24 h after bombardment. The green emission of PIN1-dendra2 was obtained by irradiating with 488-nm laser prior to photo-conversion. Setting for the green channel and the red channel was set as in Figure S2A. Photo-conversion of PIN1-dendra2 expressed in an Arabidopsis leaf PC was achieved by irradiation with 405-nm laser. (B, C) Quantitative analysis of reduction of photo-converted signal in absolute value. Datasets used for Figure 1E (B) or Figure 4B (C) were used for quantification. (TIF) [file pbio.1001299.s003.tif]

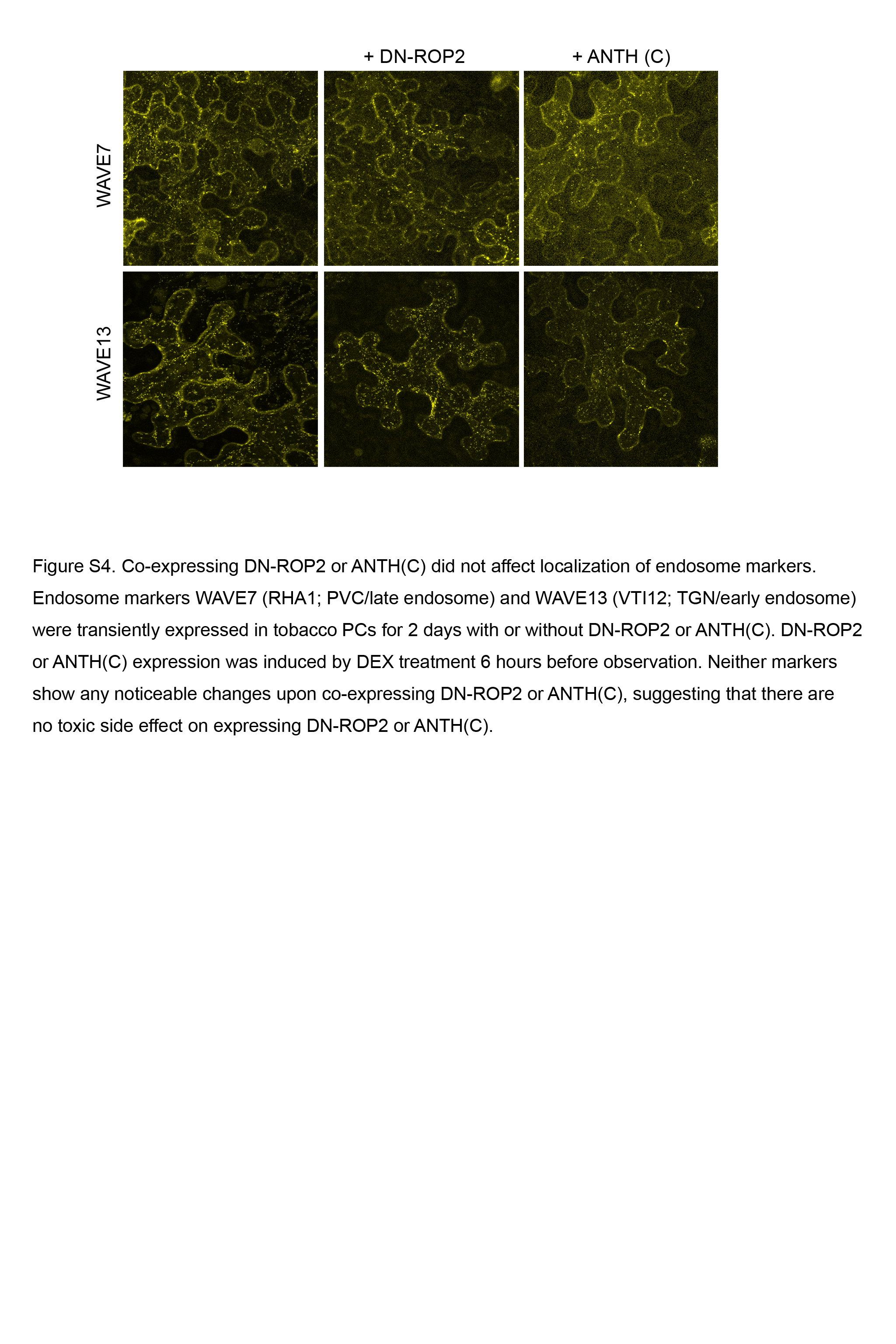

Supplement: Figure S4 — Coexpressing DN-ROP2 or ANTH(C) did not affect localization of endosome markers. Endosome markers WAVE7 (RHA1; PVC/late endosome) and WAVE13 (VTI12; TGN/early endosome) were transiently expressed in tobacco PCs for 2 d with or without DN-ROP2 or ANTH(C). DN-ROP2 or ANTH(C) expression was induced by DEX treatment 6 h before observation. Neither markers show any noticeable changes upon coexpressing DN-ROP2 or ANTH(C), suggesting that there are no toxic side effect on expressing DN-ROP2 or ANTH(C). (TIF) [file pbio.1001299.s004.tif]

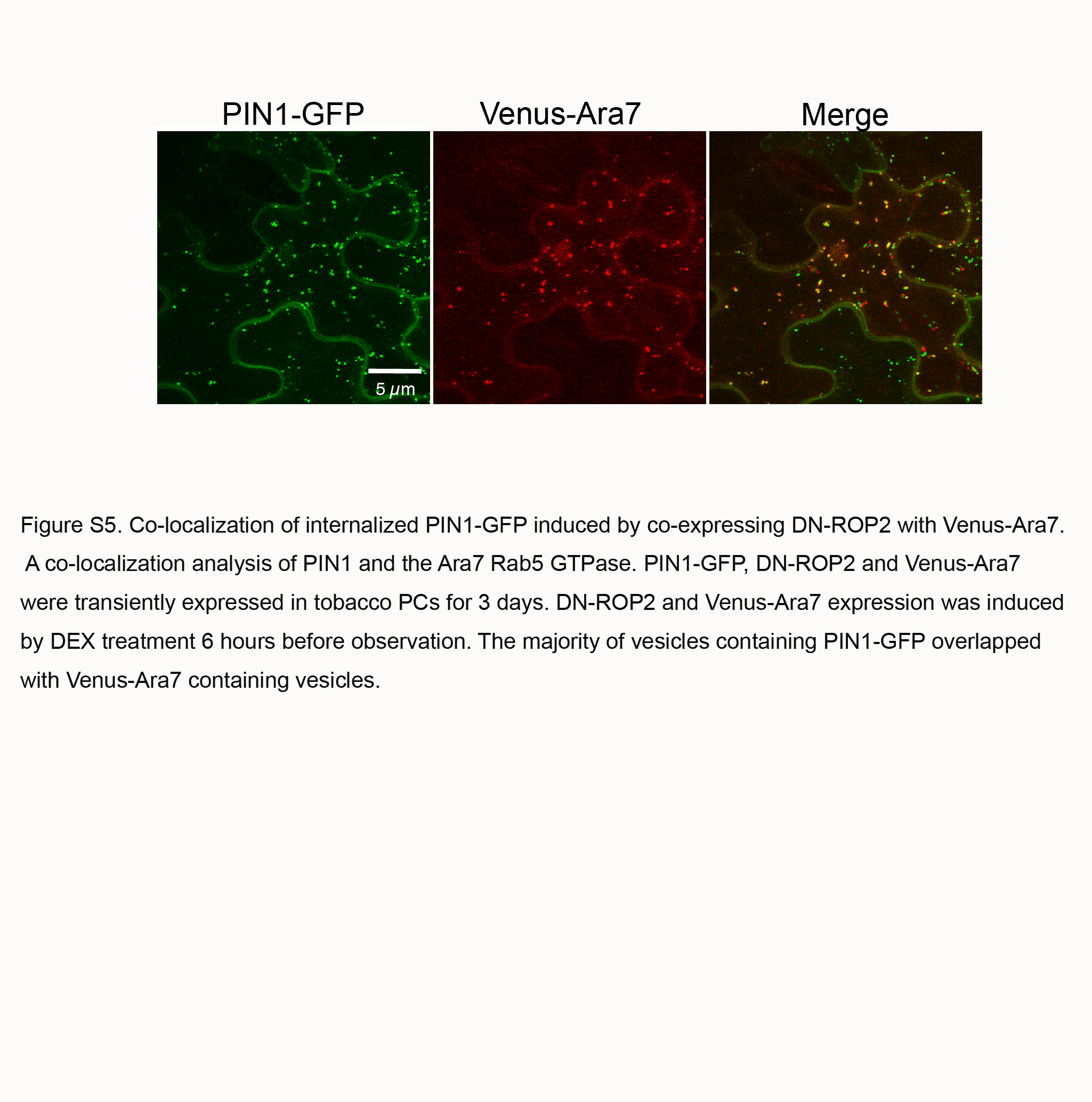

Supplement: Figure S5 — Colocalization of internalized PIN1-GFP induced by coexpressing DN-ROP2 with Venus-Ara7. A colocalization analysis of PIN1 and the Ara7 Rab5 GTPase. PIN1-GFP, DN-ROP2 and Venus-Ara7 were transiently expressed in tobacco PCs for 3 d. DN-ROP2 and Venus-Ara7 expression was induced by DEX treatment 6 h before observation. The majority of vesicles containing PIN1-GFP overlapped with Venus-Ara7 containing vesicles. (TIF) [file pbio.1001299.s005.tif]

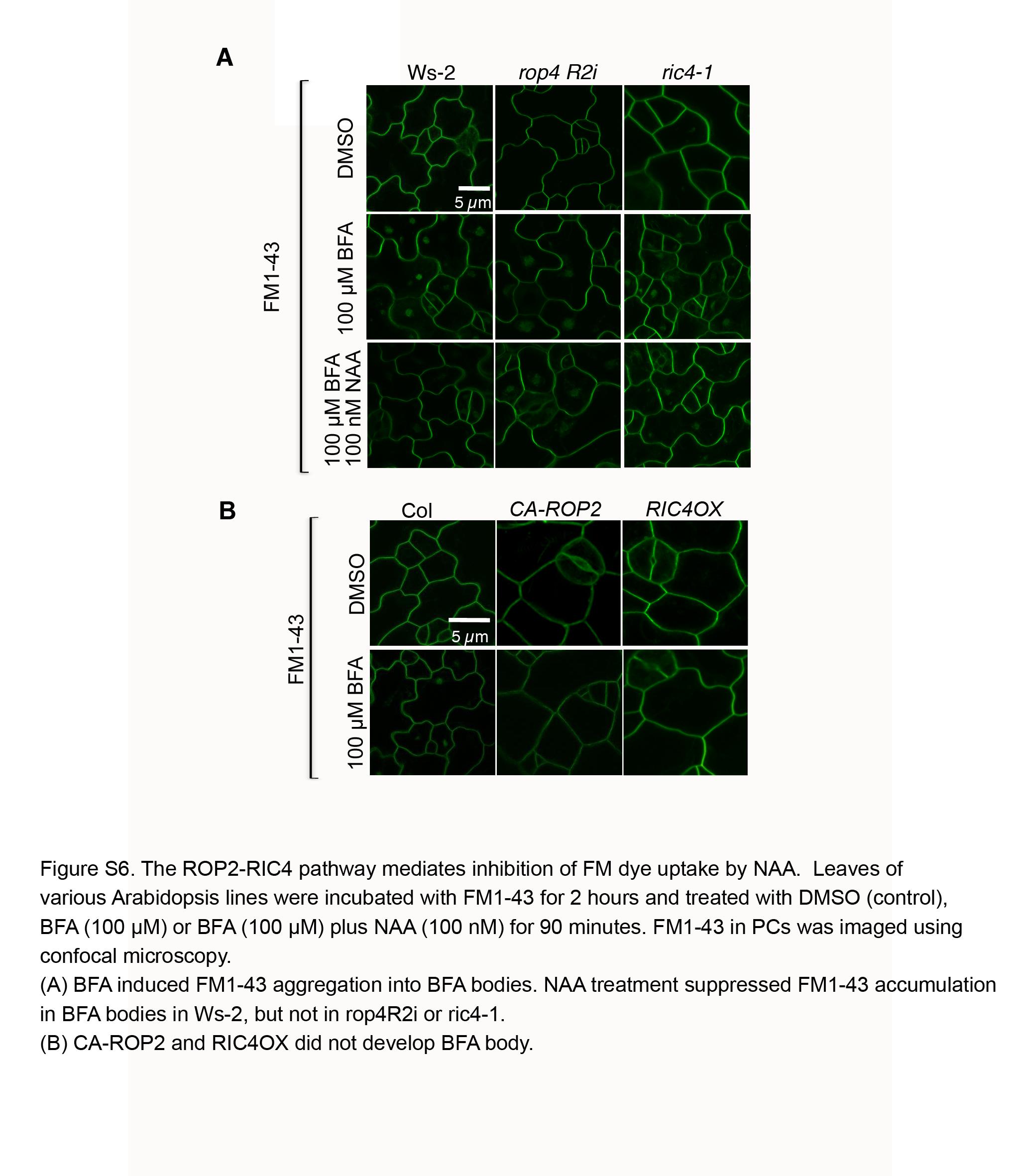

Supplement: Figure S6 — The ROP2-RIC4 pathway mediates inhibition of FM dye uptake by NAA. Leaves of various Arabidopsis lines were incubated with FM1-43 for 2 h and treated with DMSO (control), BFA (100 µM), or BFA (100 µM) plus NAA (100 nM) for 90 min. FM1-43 in PCs was imaged using confocal microscopy. (A) BFA induced FM1-43 aggregation into BFA bodies. NAA treatment suppressed FM1-43 accumulation in BFA bodies in Ws-2, but not in rop4R2i or ric4-1. (B) CA-ROP2 and RIC4OX did not develop BFA body. (TIF) [file pbio.1001299.s006.tif]

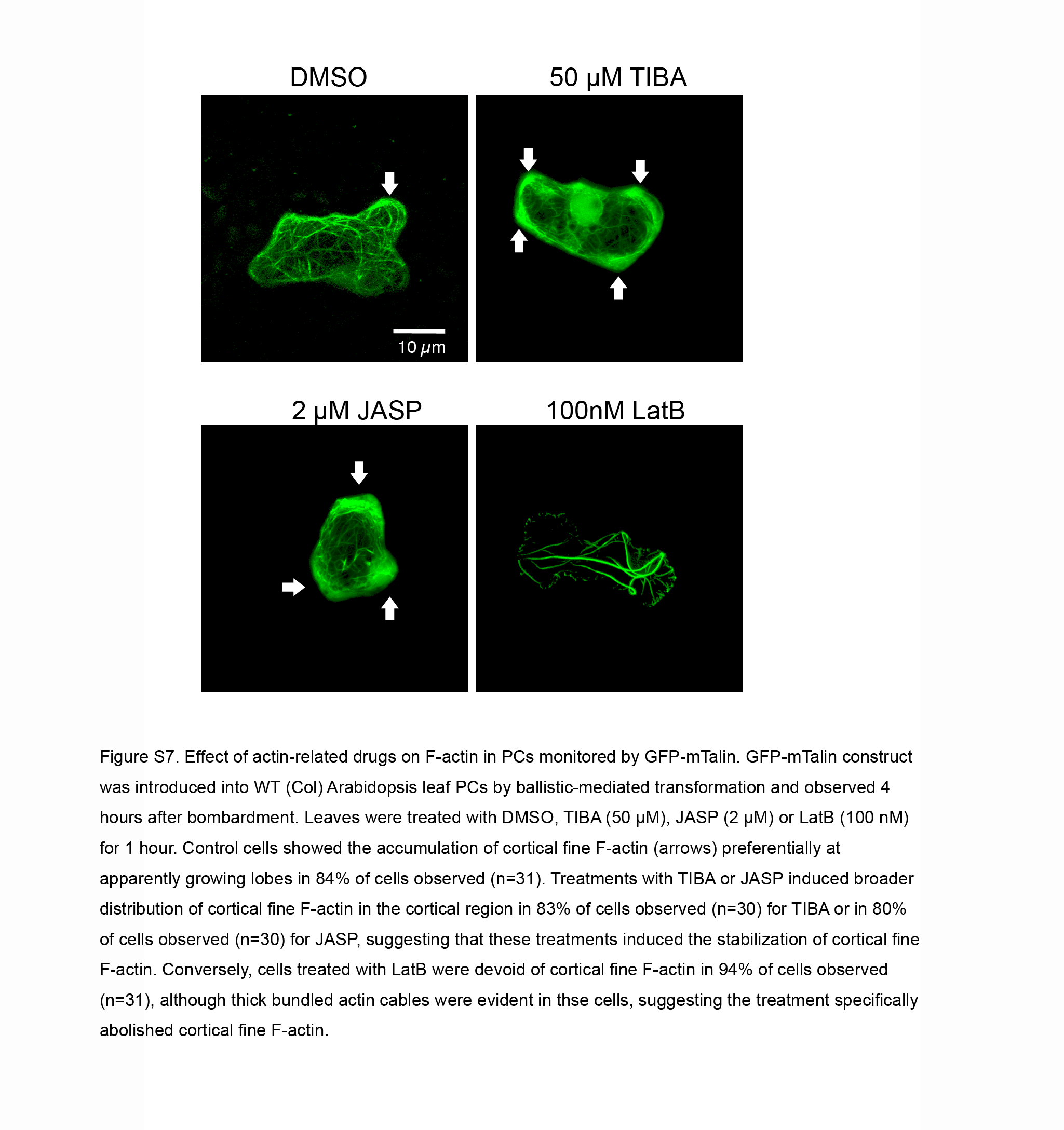

Supplement: Figure S7 — Effect of actin-related drugs on F-actin in PCs monitored by GFP-mTalin. GFP-mTalin construct was introduced into WT (Col) Arabidopsis leaf PCs by ballistic-mediated transformation and observed 4 h after bombardment. Leaves were treated with DMSO, TIBA (50 µM), JASP (2 µM), or LatB (100 nM) for 1 h. Control cells showed the accumulation of cortical fine F-actin (arrows) preferentially at apparently growing lobes in 84% of cells observed (n = 31). Treatments with TIBA or JASP induced broader distribution of cortical fine F-actin in the cortical region in 83% of cells observed (n = 30) for TIBA or in 80% of cells observed (n = 30) for JASP, suggesting that these treatments induced the stabilization of cortical fine F-actin. Conversely, cells treated with LatB were devoid of cortical fine F-actin in 94% of cells observed (n = 31), although thick bundled actin cables were evident in these cells, suggesting the treatment specifically abolished cortical fine F-actin. (TIF) [file pbio.1001299.s007.tif]

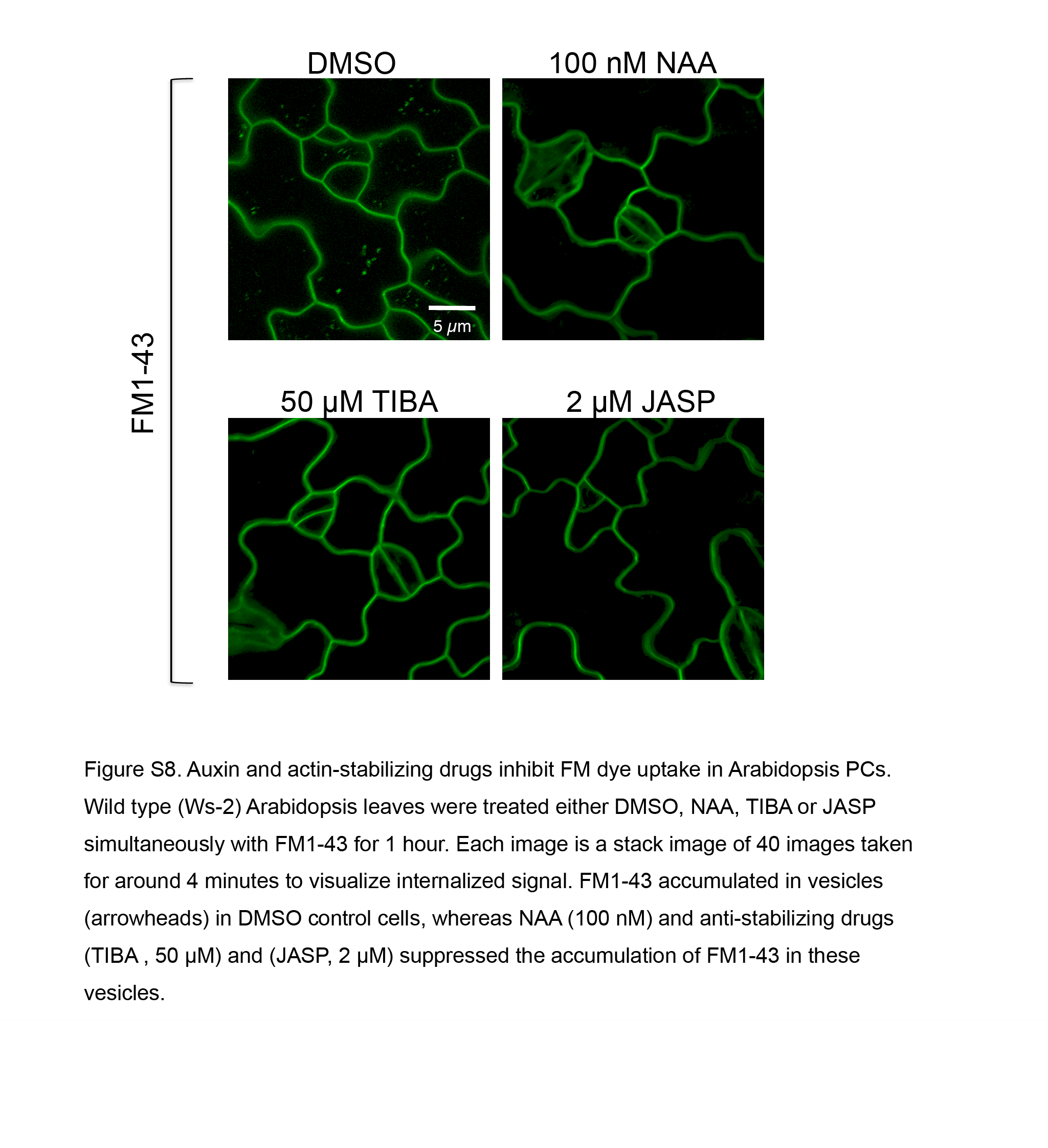

Supplement: Figure S8 — Auxin and actin-stabilizing drugs inhibit FM dye uptake in Arabidopsis PCs. WT (Ws-2) Arabidopsis leaves were treated either DMSO, NAA, TIBA, or JASP simultaneously with FM1-43 for 1 h. Each image is a stack image of 40 images taken for around 4 min to visualize internalized signal. FM1-43 accumulated in vesicles (arrowheads) in DMSO control cells, whereas NAA (100 nM) and antistabilizing drugs (TIBA, 50 µM) and (JASP, 2 µM) suppressed the accumulation of FM1-43 in these vesicles. (TIF) [file pbio.1001299.s008.tif]

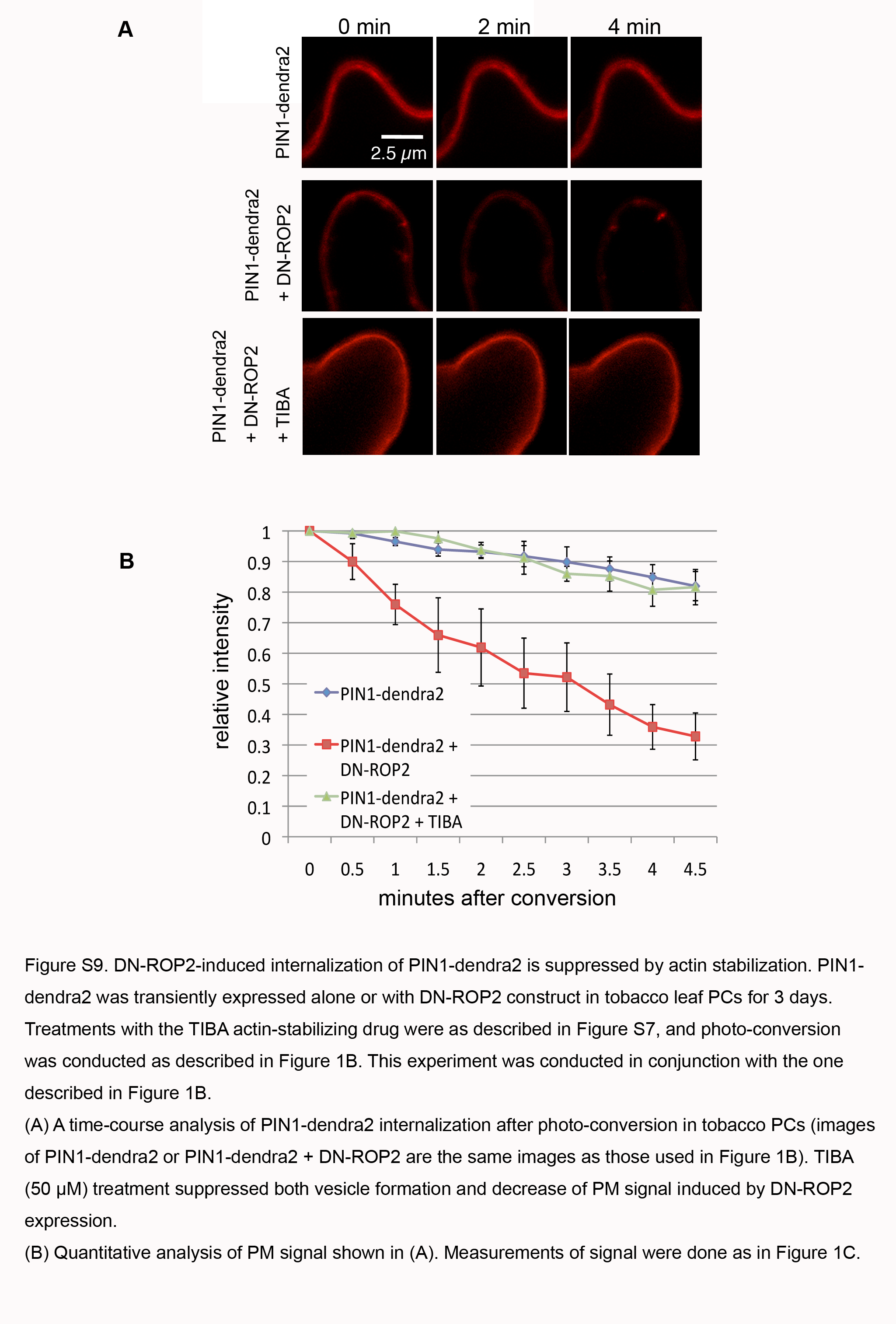

Supplement: Figure S9 — DN-ROP2–induced internalization of PIN1-dendra2 is suppressed by actin stabilization. PIN1-dendra2 was transiently expressed alone or with DN-ROP2 construct in tobacco leaf PCs for 3 d. Treatments with the TIBA actin-stabilizing drug were as described in Figure S7, and photo-conversion was conducted as described in Figure 1B. This experiment was conducted in conjunction with the one described in Figure 1B. (A) A time-course analysis of PIN1-dendra2 internalization after photo-conversion in tobacco PCs (images of PIN1-dendra2 or PIN1-dendra2+DN-ROP2 are the same images as those used in Figure 1B). TIBA (50 µM) treatment suppressed both vesicle formation and decrease of PM signal induced by DN-ROP2 expression. (B) Quantitative analysis of PM signal shown in (A). Measurements of signal were done as in Figure 1C. (TIF) [file pbio.1001299.s009.tif]
